# Infrared thermography in the detection of arthritis: a systematic review of diagnostic performance compared with joint ultrasound

**DOI:** 10.1007/s00296-026-06093-5

**Published:** 2026-03-12

**Authors:** Julian Bastelberger, Marina Clasen, Christian Geber, Martin Offenbächer, Rebecca Hasseli, Gabriel Dias Rodrigues, Andreas Schwarting, Konstantinos Triantafyllias

**Affiliations:** 1 Department of Rheumatology, Acute Rheumatology Center, Rhineland-Palatinate, Bad Kreuznach, Germany; 2 General Medical Practice, Praxis Busch/Reeh, Oppenheim, Germany; 3 Deutsches Schmerz-Zentrum, Mainz, Germany; 4 Department of Rehabilitation Medicine for Rheumatology and Orthopaedics, Klinikum Bad Gastein, Bad Gastein, Austria; 5 Section of Rheumatology and Clinical Immunology, Department of Internal Medicine D, University Hospital Münster, Münster, Germany; 6 4Health Lab, Department of Physiology and Pharmacology, Federal Fluminense University, Niterói, Rio de Janeiro, Brazil; 7 Department of Internal Medicine I, Division of Rheumatology and Clinical Immunology, Johannes Gutenberg University Medical Center, Langenbeckstraße 1, 55131 Mainz, Germany

**Keywords:** Thermography, Ultrasonography, Joint, Arthritis, Rheumatoid arthritis, Psoriatic arthritis

## Abstract

Early detection of arthritis in autoimmune rheumatic diseases (ARDs) is critical to prevent irreversible damage. Joint ultrasound (US) offers high sensitivity and availability in a routine clinical practice. However, US is limited by examiner dependency and resource requirements. Infrared thermography (IRT) is a non-invasive, radiation-free method to examine surface temperature alterations linked to arthritis. Although promising, its diagnostic performance relative to joint US remains incompletely defined. The aim of this review was to examine the literature on IRT and its relationship to joint US. We conducted a systematic review of PubMed, Web of Science, Directory of Open Access Journals (DOAJ) and Cochrane Central Register of Controlled Trials (CENTRAL) for studies published between January 2000 and December 2025. Studies were included if they assessed arthritic conditions using both IRT and US. Data on patient cohorts, assessment methods, findings, and diagnostic accuracy were extracted. Of 945 records, 19 studies met the inclusion criteria, primarily in rheumatoid arthritis (*n* = 13) and mixed populations (*n* = 4). IRT consistently differentiated inflamed from healthy joints. Sensitivity detecting arthritis ranged from 79 to 100%, specificity from 51 to 94%. Methods varied from basic temperature measures to advanced approaches, including algorithmic segmentation and machine learning-based scores such as ThermoJIS and ThermoDAI. Despite methodological differences, IRT demonstrated reproducible results and was particularly effective in detecting subclinical synovitis. IRT shows strong potential as a complementary, examiner-independent tool for detecting and monitoring joint inflammation in ARDs. Its consistent correlation with US suggests that IRT could serve as a useful adjunct in rheumatologic diagnostics. Future studies with standardized protocols are needed to establish its clinical utility.

## Introduction

Early diagnosis and targeted management of autoimmune rheumatic diseases (ARD) such as rheumatoid arthritis (RA), psoriatic arthritis (PsA), and spondyloarthritis (SpA) are crucial for preventing inflammation-driven joint and systemic damage [1–8]. Without timely treatment, these conditions can lead to significant disability, increased morbidity, and even mortality [1, 9–12]. ARDs have a profound impact on patients’ quality of life [13, 14] and implications for their families [15].

While the precise etiology and pathophysiological mechanisms of inflammatory rheumatic diseases are still being studied, current evidence points to a multifactorial process in which autoreactive B and T cells play a central role, driving acute inflammation and subsequent tissue damage [16]. This inflammatory response is characterized by the following cardinal signs -*rubor*, *calor*, *dolor*, *tumor*, and *functio laesa* [17]. A key pathophysiological feature is the increase in local blood flow, leading to elevated temperature in affected regions, which can often be detected during clinical examination [18]. However, even subtle or imperceptible changes in joint surface temperature may reflect disease exacerbations and ongoing inflammatory activity [19, 20]. Such changes can be perceived through palpation but are more accurately quantified using infrared medical thermography [21].

Infrared Thermography (IRT) is an effective tool that can make marginal temperature fluctuations visible. Modern, high-resolution infrared cameras are used to visualize the temperature of individual sections of an examined region on a screen using color coding [19, 22–24]. Infrared radiation is detected using moisture-sensitive salts such as germanium or zinc selenide, as well as microbolometer detectors [25]. This technology was originally used for military or technical purposes and became increasingly popular in medicine in the late 1950s, 1960s and 1970s [25]. The so-called *pyroscan*, a British prototype system, was utilized in 1959 to measure increased temperature of an arthritic joint on the basis of the first electronic sensor for infrared radiation [23, 26]. By the 1970s, studies had demonstrated a correlation between the surface temperature of arthritic joints, intra-articular inflammation, and specific biomarkers [27, 28]. Furthermore, IRT was able to effectively differentiate arthritic from healthy joints [29].

As a non-invasive, non-ionizing, and reliably reproducible technique, IRT holds significant potential for diagnostic applications [30]. The technology has been explored in a variety of medical contexts, including the early detection of malignancies such breast cancer [31–33] and skin cancer [34, 35], fever screening [36], assessment of diabetic foot complications [37, 38], peripheral artery disease [39, 40], and the evaluation of joint pathologies such as arthritis [41, 42]. In addition, IRT can assist in confirming Raynaud’s phenomenon and, when combined with thermal challenges, differentiating primary from secondary forms. In systemic sclerosis, it also shows promise as a noninvasive tool for assessing disease activity and monitoring therapeutic response [43–46].

In Rheumatology, conventional diagnostic modalities have various limitations. X-rays expose patients to radiation and provide limited information regarding acute inflammatory joint changes [47], while magnetic resonance imaging (MRI) is costly, time-consuming, and not feasible in every clinical setting [48, 49]. Joint ultrasound (US) offers many advantages including high sensitivity and availability, but also has notable drawbacks, such as its high dependence on examiner expertise and the need for significant personnel resources [50–57]. Newer imaging techniques, including optical spectral transmission (OST) [58–66] and fluorescence optical imaging (FOI) [67, 68], have shown promise in detecting early joint inflammation and vascular changes, but their clinical utility has not yet been fully established. In contrast, IRT is an examiner-independent, non-invasive, and relatively inexpensive method that may complement these modalities, providing a rapid assessment of inflammatory activity for both initial diagnosis and follow-up [41, 69–71]. This approach may also be applicable in addition to a telemedicine-based assessments [72, 73] or to facilitate AI-assisted patient care within automated diagnostic or therapeutic workflows [74, 75].

The role of infrared thermography (IRT) in rheumatologic diagnostics has been explored in several studies, and interest in this emerging technique continues to expand in the past years [41, 71, 76, 77]. However, data comparing it to reference diagnostic techniques such as MRI or joint ultrasound (US) remain significantly more limited [71]. Although recent research has shown promising results [78, 79] these have not been collectively examined or synthesized systematically until now. This constitutes a significant gap in the scientific literature, as comparative assessments with well-established imaging modalities are essential to determine the true diagnostic value, reliability, and clinical applicability of IRT.

The aim of this review was therefore to collate and analyze the published literature on this topic, systematically examining the potential and limitations of IRT in rheumatological diagnostics.

To our knowledge, this is the first systematic review focusing specifically on studies evaluating IRT alongside joint US.

## Methods

This systematic review was conducted in accordance with the PRISMA (Preferred Reporting Items for Systematic Reviews and Meta-Analyses) guidelines [80]. The aim was to analyze the diagnostic benefits of IRT in comparison to other imaging techniques in inflammatory and degenerative joint diseases, and to classify the existing literature on this topic.

The literature search was conducted in the PubMed database (December 28, 2025), Web of Science (WoS) database (December 28, 2025) and Directory of Open Access Journals (DOAJ; January 2, 2026) and Cochrane Central Register of Controlled Trials (CENTRAL; January 2, 2026). The search strategy combined keywords and MeSH terms on the topics of thermography, various joint diseases, and diagnostic imaging, like joint US, magnetic resonance imaging (MRI), and bone scintigraphy (specific search strings in the Table 1, adapted search grammar for WoS, DOAJ and CENTRAL). The search periods covered publications from January 1, 2000, to December 28, 2025. The articles under consideration were in English or German.

**Table 1:** Complete search strategy in PubMed, Web of Science (WoS), Directory of Open Access Journals (DOAJ), and Cochrane Central Register of Controlled Trials (CENTRAL, showing search terms, filters (2000–2025), and date of last access

Database and last access	Search terms and filters used between 2000 and 2025
PubMed (December 28, 2025)	(thermography[tiab] OR "thermal imaging"[tiab] OR "thermal image"[tiab] OR "infrared imaging"[tiab] OR "infrared image"[tiab] OR "infrared thermography"[tiab] OR "infrared camera"[tiab]) AND (arthritis[tiab] OR arthritides[tiab] OR arthropathy[tiab] OR arthropathies[tiab] osteoarthritis[tiab] OR "degenerative joint disease"[tiab] OR "degenerative joint diseases"[tiab] OR "rheumatoid arthritis"[tiab] OR "spondylarthritis"[tiab] OR "ankylosing spondylitis"[tiab] OR "systemic sclerosis"[tiab] OR lupus[tiab] OR "connective tissue disease"[tiab] OR "connective tissue diseases"[tiab] OR "inflammatory joint disease"[tiab] OR "inflammatory joint diseases"[tiab] OR gout[tiab] OR "Gout"[MeSH Terms] OR pseudogout[tiab] OR chondrocalcinosis[tiab] OR "Calcium Pyrophosphate"[MeSH Terms] OR "chondrocalcinosis articularis"[tiab] OR "reactive arthritis"[tiab] OR "reactive arthritides"[tiab] OR "Reiter syndrome"[tiab] OR "Reactive Arthritis"[MeSH Terms] OR "Reiter Syndrome"[MeSH Terms] OR "psoriatic arthritis"[tiab] OR juvenile idiopathic arthritis[tiab] OR "Arthritis, Juvenile"[MeSH Terms] OR septic arthritis[tiab] OR "Arthritis, Infectious"[MeSH Terms] OR enteropathic arthritis[tiab] OR inflammatory arthritis[tiab] OR "Arthritis"[MeSH Terms] OR "Rheumatoid Arthritis"[MeSH Terms] OR "Osteoarthritis"[MeSH Terms] OR "Spondylarthropathies"[MeSH Terms] OR "Collagen Diseases"[MeSH Terms] OR "Connective Tissue Diseases"[MeSH Terms]) AND (imaging[tiab] OR MRI[tiab] OR "magnetic resonance"[tiab] OR ultrasounds[tiab] OR ultrasound[tiab] OR sonography[tiab] OR sonographies[tiab] OR "musculoskeletal ultrasound"[tiab] OR "nuclear imaging"[tiab] OR scintigraphy[tiab] OR "radiological imaging"[tiab] OR "Ultrasonography"[MeSH Terms] OR "Magnetic Resonance Imaging"[MeSH Terms] OR "Diagnostic Imaging"[MeSH Terms])
Web of Science (December 28, 2025)	(TS = (thermography OR "thermal imaging" OR "thermal image" OR "infrared imaging" OR "infrared image" OR "infrared thermography" OR "infrared camera") AND TS = (arthritis OR arthritides OR arthropathy OR arthropathies OR osteoarthritis OR "degenerative joint disease" OR "degenerative joint diseases" OR "rheumatoid arthritis" OR spondylarthritis OR "ankylosing spondylitis" OR "systemic sclerosis" OR lupus OR "connective tissue disease" OR "connective tissue diseases" OR "inflammatory joint disease" OR "inflammatory joint diseases" OR gout OR pseudogout OR chondrocalcinosis OR "chondrocalcinosis articularis" OR "reactive arthritis" OR "reactive arthritides" OR "Reiter syndrome" OR "psoriatic arthritis" OR "juvenile idiopathic arthritis" OR "septic arthritis" OR "enteropathic arthritis" OR "inflammatory arthritis" OR "collagen diseases") AND TS = (imaging OR MRI OR "magnetic resonance" OR ultrasounds OR ultrasound OR sonography OR sonographies OR "musculoskeletal ultrasound" OR "nuclear imaging" OR scintigraphy OR "radiological imaging")) AND PY = (2000–2025)
Directory of Open Access Journals (DOAJ, January 2, 2026)	"thermography" OR "thermal imaging" OR "infrared imaging" AND arthritis OR osteoarthritis OR "rheumatoid arthritis" OR "psoriatic arthritis" OR gout OR "rheumatoid arthritis" OR spondylarthritis OR "ankylosing spondylitis" OR lupus OR "connective tissue disease" OR "inflammatory arthritis" OR gout OR pseudogout OR chondrocalcinosis OR "reactive arthritis" OR "Reiter syndrome" OR "psoriatic arthritis" OR "juvenile idiopathic arthritis" OR "septic arthritis" OR "enteropathic arthritis" OR imaging OR MRI OR "magnetic resonance" OR ultrasound OR sonography OR "musculoskeletal ultrasound" OR scintigraphy OR "radiological imaging"
Cochrane Central Register of Controlled Trials (CENTRAL) (January 2, 2026)	(thermography OR "thermal imaging" OR "thermal image" OR "infrared imaging" OR "infrared image" OR "infrared thermography" OR "infrared camera") AND (arthritis OR arthritides OR arthropathy OR arthropathies OR osteoarthritis OR "degenerative joint disease" OR "degenerative joint diseases" OR "rheumatoid arthritis" OR spondylarthritis OR "ankylosing spondylitis" OR "systemic sclerosis" OR lupus OR "connective tissue disease" OR "connective tissue diseases" OR "inflammatory joint disease" OR "inflammatory joint diseases" OR gout OR pseudogout OR chondrocalcinosis OR "chondrocalcinosis articularis" OR "reactive arthritis" OR "reactive arthritides" OR "Reiter syndrome" OR "psoriatic arthritis" OR "juvenile idiopathic arthritis" OR "septic arthritis" OR "enteropathic arthritis" OR "inflammatory arthritis") AND (imaging OR MRI OR "magnetic resonance" OR ultrasound OR ultrasounds OR sonography OR sonographies OR "musculoskeletal ultrasound" OR "nuclear imaging" OR scintigraphy OR "radiological imaging")

### Study selection process

**Fig. 1:**
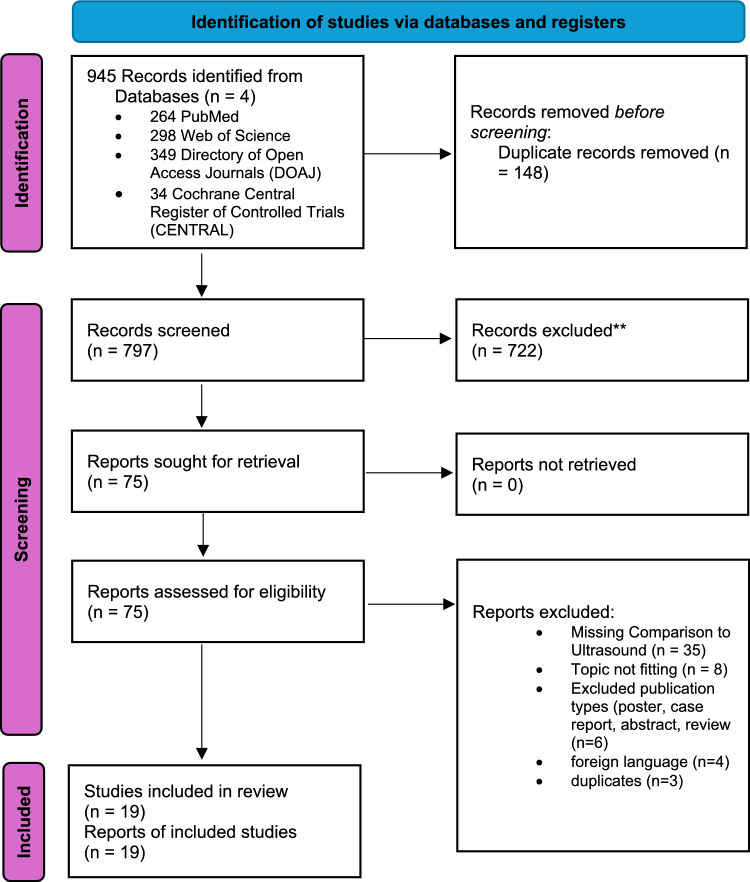
Flowchart of study identification and selection in accordance with PRISMA Guidelines [80]

The selection process involved four stages: identification, screening, eligibility assessment, and inclusion. Initial database searches yielded 945 records, which were reduced to 797 after duplicate removal. Inclusion and exclusion criteria are presented in Table 2. Titles and abstracts were then screened to identify potentially relevant studies. This was followed by a full-text analysis of the selected articles to assess their eligibility (see Fig. 1). The evaluation was performed by two independent reviewers (JB and KT). All reasons for exclusion were systematically documented. During full-text review of 75 studies, a total of 56 publications were excluded during the screening process due to the absence of a comparison with ultrasound (*n* = 35), lack of relevance to the review research question (*n* = 8), ineligible publication types (posters, case reports, abstracts, and reviews; *n* = 6), foreign language publications (*n* = 4), and duplicate records (*n* = 3). Ultimately, 19 studies were included in the final review, providing direct comparative data on thermographic and ultrasonographic assessment of joint inflammation.

**Table 2:** Inclusion and exclusion criteria, specifying population, study type, index test, comparator (reference methodology), outcomes, and language

	Inclusion	Exclusion
Population	Individuals (all age groups) with musculoskeletal complaints, suspected joint or soft tissue disorders, or pre-existing conditions associated with arthritis	Animals, in vitro studies
Study Type	Original studies comparing thermography with at least one other imaging modality (e.g., MRI, joint US) in relation to joint diseases, within the last 25 years	Case Reports, Reviews, Abstracts
Intervention / Index Test	IRT as a diagnostic tool or for disease monitoring	Studies without IRT
Comparator/ Reference Methodology	Imaging Modality like MRI or joint-US	Studies without comparison or integration of other imaging modalities
Outcomes	Measures of diagnostic accuracy (e.g., sensitivity and specificity), as well as assessments of comparability and clinical utility	Studies without findings for assessment of comparability and clinical utility
Language	English, German	Other languages

Infrared Thermography (*IRT*), Joint Ultrasound (*Joint US*), *MRI* (Magnetic Resonance Imaging)

### Data extraction

The following information was extracted for all included studies: patient population (e.g., disease, age group), comparison method (e.g., MRI, joint US), regions examined, main findings, and, where available, sensitivity and specificity values (Table 3). The results of the included studies were summarized descriptively. Similarly, information regarding the study populations was compiled (Table 4), and the various methodologies were extracted (Table 5). Additionally, an evaluation of the risk of bias in the included studies was conducted using the QUADAS-2 Assessment Tool as recommended by the PRISMA guidelines (Table 6) [80, 81].

**Table 3:** Brief description of included studies (alphabetical order), showing author and year, sample size, joint investigated, main findings, and sensitivity and specificity measures (if reported)

Author and year	Disease	Population	Joint	Main Findings	Sensitivity and specificity
Ahn et al. (2022) [82]	RA, SpA, other arthritides, OA	30	Knee	PD-positive joints showed higher Tavg, Tmax, and Tmin than PD-negative joints (all *p* < 0.05)	Tavg: sensitivity 87.5%, specificity 68.5% at cut-off 30.1 °C, AUC 0.770 (95% CI 0.581–0.959) for detecting arthritis
Capo et al. (2015) [83]	PsA	21 (10 PsA + 11 controls)	Hand	Dynamic assessment combining functional exercise and repeated IRT: PsA patients showed higher IRT parameters (*p* < 0.0025) and faster fluctuations vs. controls; Tmax correlated with PD-defined activity (small sample, qualitative conclusions only)	Not stated
Capo et al. (2018) [84]	RA	21 (10 RA + 11 controls)	Hand	RA patients showed different temperature profiles (static and dynamic, e.g., Tmax and peak during recovery) vs. PsA patients and controls (all *p* < 0.005)	Not stated
Gizinska et al. (2021) [85]	RA	130 (81 RA + 39 controls)	Dorsal side of the feet	Tavg of dorsal feet higher in RA patients vs. controls (*p* < 0.05); joint-US inflammation not consistently associated with higher Tavg (except two joints, *p* < 0.05)	Not stated
Lerkvaleekul et al. (2017) [86]	JIA	61 (46 JIA + 15 controls)	Wrist	Tmax and Tavg in ROI higher in active arthritis vs. inactive patients and controls (*p* < 0.05); ROC analysis for Tavg and joint US showed comparable results (AUC Tavg 0.93, AUC US 0.87)	Tavg cut-off > 31 °C: sensitivity 85.7%, specificity 80.0%, AUC 0.93 for detecting moderate–severe arthritis
Morales-Ivorra et al. (2022)—Study 1 [78]	RA, PsA, undifferentiated Arthritis, OA	Mixed Development cohort: 449 subjects; validation cohort: 146 RA patients	Hand	ThermoJIS (Machine learning based) showed moderate correlations with GS abnormalities (*r* = 0.49, *p* < 0.001) and PD-US (r*r*= 0.51, *p* < 0.001); identified active synovitis in symptomatic and asymptomatic patients (AUC 0.78)	ThermoJIS cut-off > 3.56: sensitivity 94%, specificity 51%, AUC 0.78 (95% CI 0.71–0.86, *p* < 0.001)
Morales-Ivorra et al. (2022)- Study 2 [87]	RA	Same Validation cohort from Study 1 (see above)	Hand	Combined activity score ThermoDAI-CRP (ThermoJIS + PGA + CRP) correlated strongly with PD abnormalities (Spearman *r*= 0.56, *p* < 0.001)	ThermoDAI-CRP sensitivity and specificity detecting active synovitis (88% and 35%), AUC not stated
Tan et al. (2020) [88]	RA	37 RA	Wrist- and Finger Joints	Significant differences in Tmax, Tmin, Tavg, and Tmax–Tmin range between PD-positive and PD-negative joints (all *p* < 0.001); corresponding results for GS findings (all *p* < 0.001)	Not stated
Tan et al. (2021) [89]	RA	37 RA	Wrist-and Finger Joints	Combined IRT and US scores correlated with disease activity (DAS28), e.g., Pearson r MAX (PD) 0.393 (95% CI 0.079–0.636, *p* = 0.016)	Not stated
Tan et al. (2024) [90]	RA	70 RA	Wrist	Swollen wrists showed higher Tmax, Tmin, Tavg, and greater US-detected joint inflammation (GS and PD) vs. unswollen joints (all *p* < 0.05)	Not stated
Tan et al. (2024) [91]	RA	87 RA	Wrist	Symptomatic swollen/tender wrists showed higher thermographic parameters vs. asymptomatic joints (*p* < 0.05); mirrored by joint-US synovitis (*p* < 0.05); subclinical activity in asymptomatic wrists correlated with higher IRT parameters (Spearman *r* PD 0.43–0.48, *p* < 0.001)	Not stated
Tan et al. (2024) [92]	RA	42 RA	MCP	Joints with active/subclinical synovitis (PD and/or GS positive) correlated with higher Tmin, Tavg, Tmax (*p* = 0.001–0.0012; Spearman *r*= 0.383–0.424)	Not stated
Tan et al. (2024) [93]	RA	80 RA	MCPs, PIP, Wrist, Elbow, Knee	Patients with PD-positive joints showed higher total temperature scores in clinically quiescent joints (*p* < 0.05)	ROC for total Tmax detecting PD positivity: AUC 0.810 (95% CI 0.687–0.933), sensitivity 95.7%, specificity 54.5%
Tan et al. (2024) [94]	RA	30 RA	Elbow	Both elbows showed significant correlation between cumulative thermographic scores and PD score (Spearman *r* = 0.40–0.55, *p*< 0.05)	Not stated
Tan et al (2025) [95]	RA	95 RA	Knee	Higher Tmax, Tmin, and Tavg correlated with GS (*r* = 0.24–0.29, *p* < 0.05) and PD (*r*= 0.21–0.43, *p* < 0.05) joint inflammation	ROC analysis of Tmax, Tmin, Tavg for detecting PD score > 0: AUC 0.63–0.82
Tan et al. (2025) [96]	RA	81 RA	MCPs	Higher summed Tmax, Tmin, Tavg correlated with GS (*r*r = 0.27–0.49, *p* < 0.05) and PD (*r*= 0.45–0.52, *p* < 0.05) joint inflammation	ROC analysis of summed thermographic temperatures for predicting summed PD score ≥ 1 of bilateral MCPs: AUC 0.80–0.82
Triantafyllias et al. (2024) [79]	RA, PsA, SpA, and Gout	(145) 75 patients with mixed disease spectrum and 70 controls	Fingers and wrists, elbows, knees, and ankles	New thermographic parameter HRR (Hotspot/ROI Ratio) differentiates inflamed vs. non-inflamed joints (*p* < 0.01) and grades of hypervascularity (*p* < 0.05)	Wrist synovitis: ROC AUC 0.91 (95% CI 0.84–0.98), sensitivity 0.83, specificity 0.88; all joints: AUC 0.76 (95% CI 0.699–0.818), sensitivity 79%, specificity 65%
Umapathy et al. (2020) [97]	RA	60 (30 RA patients and 30 controls)	Knee	PD abnormalities and higher IRT parameters correlated with ESR, CRP, DAS28, and HAQ (all *p*< 0.01)	Not stated
Vasdev et al. (2023) [98]	RA	100 (50 RA + 50 controls)	Knee	RA subjects showed higher Tavg and mean knee/thigh temperature-differential vs. controls (*p* < 0.05)	ROC analysis: AUC 0.998 (*p* = 0.001), sensitivity 100% (95% CI 92.5–100%), specificity 94.35% (95% CI 84.3–98.8%)

*ARD* (Autoimmune rheumatic diseases), *AUC* (Area under the curve), *CRP* (C-reactive Protein), *DAS28* (Disease Activity Score 28), *ESR* (Erythrocyte Sedimentation Rate), *GS* (Grey-scale ultrasound), *HAQ* (Health Assessment Questionnaire), *IRT* (Infrared Thermography), *JIA* (Juvenile Idiopathic Arthritis), *MCP* (Metacarpophalangeal joint), *OA* (Osteoarthritis), *PD* (Power doppler ultrasound), *PGA* (patient’s assessment of disease activity), *PIP* (Proximal interphalangeal joint) *PsA* (Psoriatic Arthritis), *RA* (Rheumatoid Arthritis), *ROC* (Receiver operating characteristics), *ROI* (Region of interest), *SpA* (Spondylarthritis), *Tavg* (Average Temperature), *Tmax* (Maximum Temperature), *Tmin* (Minimum Temperature), *US* (Ultrasound)

**Table 4:** Description of study populations (alphabetical order), including number of subjects, sex distribution (male/female), mean age, disease, duration of disease (years), and medication

Study	Number of Subjects	Male/Female	Mean Age	Disease	Duration of Disease in years	Medication
Ahn et al. (2022) [82]	30	11/19	51.6	RA, SpA, other arthritides, OA	Not stated	Patients on DMARDs: MTX, HCQ, SSZ, and/or biologics 26.7% of patients (8/30) on corticosteroids
Capo et al. (2015) [83]	21	11/10	51.5	PsA	Not stated	All subjects were free of any medication
Capo et al. (2018) [84]	21	8/13	52	RA	Not stated	Not stated
Gizinska et al. (2021) [85]	130	Not stated	57	RA	13	Not stated
Lerkva-leekul et al. (2017) [86]	61	Not stated	Median of different groups (inactive/arthritis) 7.7 and 10.2	JIA	Median of different groups (inactive/arthritis) 3.9 and 5.7	Not stated
Morales-Ivorra et al. (2022) Study 1 [78]	Mixed development cohort: 449 subjects, and validation cohort: 146 subjects with RA	28/118	57 in validation set	RA, PsA, undifferentiated Arthritis, OA	Not stated	Not stated
Morales-Ivorra et al. (2022) Study 2 [87]	Same Validation cohort 146 subjects with RA	28/118	57	RA	Not stated	Not stated
Tan et al. (2020) [88]	37	9/28	56.5	RA	2.5	26 (70.3%) patients were on prednisolone while 31 patients (83.8%) were on one or more DMARDs including MTX, HCQ or SSZ and/or tofacitinib
Tan et al. (2021) [89]	37, same Cohort as above	9/28	56.5	RA	2.5	70.3% of patients (26/37) on prednisolone 83.8% of patients (31/37) on ≥ 1 DMARD: MTX, SSZ, HCQ, or tofacitinib
Tan et al. (2024) [90]	70	13/57	58.5	RA	1.9	67.1% of subjects (7/70) on oral prednisolone All patients on ≥ 1 DMARD: MTX, SSZ, HCQ, and/or LEF
Tan et al. (2024) [91]	87	18/69	58.2	RA	2.25	64.4% of patients (56/87) on oral prednisolone; mean dose 4.6 ± 2.1 mg/day All patients on ≥ 1 DMARD: LEF, MTX, SSZ, and/or HCQ
Tan et al. (2024) [92]	42	7/35	57.7	RA	3.16	59.5% of patients (25/42) on oral prednisolone 100% of patients on ≥ 1 conventional DMARD: MTX, LEF, SSZ, and/or HCQ
Tan et al. (2024) [93]	80	19/61	57	RA	0.6	66.3% of patients (53/80) on oral prednisolone All patients on ≥ 1 DMARD: MTX, LEF, SSZ, and/or HCQ
Tan et al. (2024) [94]	30	7/23	57.7	RA	0.65	70% of patients (21/30) on prednisolone All patients on ≥ 1 conventional DMARD: MTX, SSZ, HCQ, and/or LEF
Tan et al. (2025) [95]	95	24/71	56.7	RA	0.65	69.5% of patients (66/95) on prednisolone All patients on ≥ 1 conventional DMARD: MTX, SSZ, HCQ, and/or LEF
Tan et al. (2025) [96]	81	22/59	54.9	RA	0,61	Not published in the original Article
Triantafyllias et al. (2024) [79]	145	42/103	53.5	RA, PsA, SpA, and Gout	Not stated	Not stated
Umapathy et al. (2020) [97]	60	Not stated	46	RA	Not stated	Not stated
Vasdev et al. (2023) [98]	100	21/79	44.71	RA	More than 80% less then 10 years of disease duration	Not stated

*DMARD* (Disease-modifying Drug), *HCQ* (Hydroxychloroquine), IRT (Infrared Thermography), *JIA* (Juvenile Idiopathic Arthritis), *LEF* (Leflunomide) *MTX* (Methotrexate), *OA* (Osteoarthritis), *PsA* (Psoriatic Arthritis), *RA* (Rheumatoid Arthritis), *SpA* (Spondylarthritis), *SSZ* (Sulfasalazine)

**Table 5:** Brief description of methodology of included studies, including preparation of measurement/environmental conditions, thermographic parameters, camera and its positioning, manual/automatic ROI selection (and protocol if applicable), processed parameters and notable features and the reference test method/protocol

Study	Preparation of the Measurement/Environmental Conditions	Thermographic Parameters	Camera	Manual/Automatic ROI-Selection	Processed Parameters and notable Features	Methods/Protocoll of Reference test
Ahn et al. (2022) [82]	Participants assessed after 20 min acclimatization (22 °C ± 1 °C, 55% humidity, stable airflow)	Tmax, Tmin, Tavg, Trange	FLIR ONE Pro camera Tripod stabilization, 1 m camera-to-knee distance	Manual ROI: circular knee ROI (≥ 5.5 cm) covering medial/lateral femoral epicondyles at patellar center	–-	PD signal in parapatellar recess assessed per EULAR guidelines [99–101]
Capo et al. (2015) [83]	IRT in controlled room (23 °C, 50–60% humidity, no direct ventilation) after 20 min acclimatization	Multiple thermographic parameters analyzed at dynamic points and timespans	FLIR SC660 QWIP Fixed 50 cm distance to participants’ hands	Manual ROI: PIP, DIP, MCP joints, nails, interosseous muscles of dominant hand	RT images at baseline (1.5 min) and after 2 min functional exercise with repeated isometric contractions	PD-Joint US per EULAR/OMERACT guidelines [99–101]
Capo et al. (2018) [84]	IRT in controlled room (23 °C, 50–60% humidity, no direct ventilation) after 20 min acclimatization	Multiple thermographic parameters analyzed at dynamic points and timespans	FLIR SC660 QWIP Fixed 50 cm distance to participants’ hands	Manual ROI: PIP, DIP, MCP joints, nails, interosseous muscles of dominant hand hand	RT images at baseline (1.5 min) and after 2 min functional exercise with repeated isometric contractions using a calibrated dynamometer	PD-Joint US per EULAR/OMERACT guidelines [99–101]
Gizinska et al. (2021) [85]	Participants avoided stimulants, non-prescribed drugs, intensive activity; 20 min acclimatization at 21 °C, 40 ± 10% humidity, draught-free, no sunlight following European Association of Thermology guidelines [102, 103]	T avg	ThermaCAM SC640 (FLIR) tripod 50 cm above feet, perpendicular; dorsal foot images captured	Manual ROI following the Glamorgan protocol [104]	–	Dorsal foot (MTP 1–5) examined; inflammation = minimal synovial hypertrophy ± mild increased vascularization [105, 106]
Lerkva-leekul, B. et al. (2017) [86]	IRT in draught-free room, 22 ± 0.5 °C, 50 ± 10% humidity	T avg und T max and Heat Distribition Index (HDI, Salisbury 1983 [29]	FLIR E60 1 m from wrist, anterior view	Manual ROI: rectangle on 3rd metacarpal with ulna as medial and radius lateral boundaries	–-	PD was performed using the lowest pulse repetition without background noise [100, 101]. Grey-scale US was graded on a 4-point semi-quantitative scale [107]
Morales-Ivorra, I., et al. (2022) Study 1 [78]	IRT without acclimatization or controlled conditions	ThermoJIS scores generated via machine learning, IRT features associated with PD sum score of patient’s hand	Flir One Pro or a Thermal Expert TE- Q1 camera No fixed camera-to-hand distance was used; the researcher ensured proper framing and focus of the images	Automatic ROI: algorithm-defined local areas with high multidirectional intensity variation	See Thermographic parameters	Joint US with PD was performed following EULAR and OMERACT guidelines [99–101]
Morales-Ivorra et al. (2022) Study 2 [87]	IRT without acclimatization or controlled conditions	ThermoJIS scores generated via machine learning, IRT features associated with PD sum score of patient’s hand	Thermal Expert TE- Q1 No fixed camera-to-hand distance was used; the researcher ensured proper framing and focus of the images	Automatic ROI: algorithm-defined local areas with high multidirectional intensity variation	ThermoJIS was integrated with additional clinical information	Joint US with PD was performed following EULAR and OMERACT guidelines [99–101].
Tan et al. (2020) [88]	IRT in draught-free, windowless room (~ 22 °C); ≥ 15 min acclimatization, obstructive items removed	T max, T min, T avg, T range	FLIR T640 positioned 50 cm above each hand, hand on a flat surface	Manual ROI on greyscale: rectangular boxes over wrists, MCPs, thumb IPs, PIPs (both hands)	–-	PD and GS joint inflammation graded semi-quantitatively, using Backhaus et al. definitions for PD [99] and an ultrasonographic atlas for GS grading[108]
Tan et al. (2021) [89]	IRT in draught-free, windowless room (~ 22 °C); ≥ 15 min acclimatization, obstructive items removed	T max, T min, T avg	FLIR T640 positioned 50 cm above each hand, on a flat surface	Manual ROI on greyscale: rectangular boxes over wrists, MCPs, thumb IPs, PIPs (both hands)	lowest T min among joints negative for both PD and GS inflammation was defined as individual control temperature. The difference between each joint’s T max and the control temperature was summed across 22 joints to calculate MAX IRT parameter. Equivalent procedures were applied for T min and T avg	PD and GS joint inflammation were graded semi-quantitatively, using Backhaus et al. definitions for PD [99] and an ultrasonographic atlas for GS grading[108]
Tan et al. (2024) [90]	IRT in draught-free, windowless room (~ 23 °C); ≥ 15 min acclimatization, obstructive items removed	T max, T min, T avg	FLIR T640 positioned 50 cm above each hand, on a flat surface	Manual ROI: rectangular boxes over wrists	Dorsal wrist scanned at distal radioulnar and radiocarpal/intercarpal recesses; PD and GS scores summed to Total PD (TPD) and Total GS (TGS) for right wrist	Joint US with PD was performed following EULAR and OMERACT guidelines [99–101]
Tan et al. (2024) [91]	IRT in draught-free, windowless room (~ 23 °C); ≥ 15 min acclimatization	T max, T min, T avg	FLIR T640 positioned 50 cm above each hand, on a flat surface	Manual ROI: rectangular boxes over wrists	Dorsal wrist scanned at distal radioulnar and radiocarpal/intercarpal recesses; PD and GS scores summed to Total PD (TPD) and Total GS (TGS) for right wrist	Joint US with PD was performed following EULAR and OMERACT guidelines [99–101]
Tan et al. (2024) [92]	Standardized IRT following established methods [43]. IRT in draught-free, windowless room (~ 23 °C); ≥ 15 min acclimatization [43]	T max, T min, T avg	FLIR T640 positioned 50 cm above each hand, on a flat surface	Target ROIs on greyscale images manually segmented; box over the MCPs, following commonly used methods [43]	T min, T avg, and T max at the 10 MCPs were summed (Total T min, Total T avg, Total T max). Similarly, GS and PD sub-scores summed up (Total GS (TGS) and Total PD (TPD) scores)	Joint US with PD was performed following EULAR and OMERACT guidelines [99–101]
Tan et al. (2024) [93]	Standardized IRT following established methods [43]. IRT in draught-free, windowless room (~ 23 °C); ≥ 15 min acclimatization [43]	T max, T min, T avg	FLIR T640 positioned 50 cm above each hand, on a flat surface	Manual ROI selection over e.g., wrist, MCPs	Summed thermographic temperatures (Total T max, Total T avg, Total T min) compared patients with Total PD (TPD) score > 1 versus ≤ 1	Joint US with PD was performed following EULAR and OMERACT guidelines [99–101]
Tan et al. (2024) [94]	Standardized IRT following established methods [43]. IRT in draught-free, windowless room (~ 23 °C); ≥ 15 min acclimatization [43]	T max, T min, T avg	FLIR T640 positioned 50 cm from medial, lateral, posterior, and anterior aspects of each elbow	Manual ROI: Target ROIs at the medial, lateral, posterior, and anterior aspects [43]	Composite scores MIN, MAX, AVG calculated from T min, T max, T avg of medial, lateral, posterior, anterior elbow aspects; GS and PD sub-scores summed to Total GS (TGS) and Total PD (TPD)	Joint US with PD was performed following EULAR and OMERACT guidelines [99–101]
Tan et al. (2025) [95]	Standardized IRT performed following prior established methods [71, 88]. IRT in draught-free, windowless room (~ 23 °C); ≥ 15 min acclimatization [71, 88]	T max, T min, T avg	FLIR T640 positioned 50 cm from medial, lateral and anterior aspects of the knee	Manual ROI: Target ROIs at the medial, lateral, posterior, and anterior aspects [71, 88]	–-	Joint US with PD was performed following EULAR and OMERACT guidelines [99–101]
Tan et al. (2025) [96]	Standardized IRT was performed following prior established methods [43, 71, 88]. IRT in draught-free, windowless room (~ 23 °C); ≥ 15 min acclimatization [43, 71, 88]	T max, T min, T avg	FLIR T865 positioned 50 cm above each hand, on a flat surface	Manual ROI: rectangular boxes over MCPs [88]	T max, T min, T avg at MCPJs for each hand compared with PD and GS scores	Joint US with PD was performed following EULAR and OMERACT guidelines [99–101]
Triantafyllias et al. (2024) [79]	Standardized protocol (European Thermographic Association [102, 103]), 15 min acclimatization; no physical strain (24 h); no smoking (4 h)	T max, T min, T avg. T range	VarioCam HD research 780 S/30 mm®	Manual ROI following the Glamorgan Protocol [104]	Hotspot/ROI ratio (HRR) calculated using k-means clustering to identify hottest pixel clusters; HRR defined as number of hotspot pixels divided by the total ROI pixels	PD activity was graded semi-quantitatively ([99, 105]: binary US scoring for GS pathology, abnormal when synovitis or joint effusion caused joint capsule distention [109, 110]
Umapathy et al. (2020) [97]	10 min acclimatization at 20 °C, 45–50% humidity; knees exposed before imaging	statistical features (e.g. mean, variance) were extracted from images and compared RA patients vs. controls	ThermaCAM-T400 handheld camera, in a posterior-anterior view of knee region, with a 1 m distance	IRT data automatically segmented with RIBS-algorithm (Regional Isotherm-Based Segmentation)	–-	Knees examined in four planes: supra-/infrapatellar, medial, and lateral. Effusion, perfusion, and synovial hypertrophy were assessed. Maximum colour Doppler activity was recorded, and perfusion intensity was quantified using colour fraction and was semi-quantitatively graded [101]
Vasdev et al. (2023) [98]	IRT performed in low-light, draught-free environment at ~ 23 °C. No alcohol 12 h, no smoking/tea/coffee ≥ 2 h, no physiotherapy/topical treatments 24 h; target area uncovered, 20 min rest before imaging	T avg	Testo(R) 885 infrared camera lateral knee aspect with hip and knee flexed at 90°, distance of 0.5 m	Manual Knee ROI: line from inferior patella to the posterior knee flexure, control ROI of equal size placed on lateral mid-thigh		PD activity was graded semi-quantitatively

*GS* (Grey-scale ultrasound), *HDI* (Heat Distribition Index), *HRR* (Hotspot/Region of Interest Ratio), *IRT* (Infrared Thermography), *MAX* (composite Tmax Score), *MCP* (Metacarpophalangeal joint, *MIN* (composite Tmin Score), *PD* (Power doppler ultrasound), *PIP* (Proximal interphalangeal joint), *RIBS* (Regional Isotherm-Based Segmentation), *ROI* (Region of interest), *Tavg* (Average Temperature), *TGS* (Total Grey Scale Score, composite Score), *Tmax* (Maximum Temperature), *Tmin* (Minimum Temperature), *Trange* (Tmax-Tmin), *US* (Ultrasound), *TPD* (Total Power Doppler Score, composite Score)

**Table 6:** Risk of bias and applicability concerns of included studies, assessed using the QUADAS-2 tool [81], covering patient selection, index test, reference standard, and flow and timing

Study	Risk of bias				Applicability concerns		
	Patient selection	Index test	Reference standard	Flow and timing	Patient selection	Index test	Reference standard
Ahn et al. (2022) [82]	High	Low	Low	Low	High	Low	Low
Capo et al. (2015) [83]	High	Low	Unclear	High	High	Low	Low
Capo et al. (2018) [84]	High	Low	Unclear	High	High	Low	Low
Gizinska et al. (2021) [85]	Low	Unclear	Low	Low	Low	Unclear	Low
Lerkvaleekul et al. (2017) [86]	Unclear	Low	Low	Low	Low	Low	Low
Morales-Ivorra et al. (2022) Study 1 [78]	Low	Unclear	Low	Low	Low	Low	Low
Morales-Ivorra et al. (2022) Study 2 [87]	High	Unclear	Low	Low	Low	Low	low
Tan et al. (2020) [88]	Unclear	Low	Low	Low	Low	Low	Low
Tan et al. (2021) [89]	High	Low	Low	Low	Low	Low	Low
Tan et al. (2024) [90]	Low	Low	Low	Low	Low	Low	Low
Tan et al. (2024) [91]	Low	Low	Low	Low	Low	Low	Low
Tan et al. (2024) [92]	Low	Low	Low	Low	Low	Low	Low
Tan et al. (2024) [93]	Unclear	Low	Low	Low	Low	Low	Low
Tan et al. (2024) [94]	Unclear	Low	Low	Low	Low	Low	Low
Tan et al. (2025) [95]	Low	Low	Low	Low	Low	Low	Low
Tan et al. (2025) [96]	Low	Low	Low	Low	Low	Low	Low
Triantafyllias et al. (2024) [79]	High	Low	Low	Low	High	Low	Low
Umapathy et al. (2020) [97]	Low	Low	Low	Unclear	Low	Low	low
Vasdev et al. (2023) [98]	Low	Low	Low	Unclear	Low	Low	low

*High* (High Risk of Bias), *Low* (Low Risk of Bias), *Unclear* (Unclear Risk of Bias)

We did not perform a meta-analysis due to the limited number of studies focusing on a single joint. Moreover, the available studies varied in outcome measures (Table 3), resulting from different study designs (Table 4); for example, outcome measures such as sensitivity and specificity were reported inconsistently. Additionally, many of the studies had small sample sizes, which further hinders direct comparison (Tables 3 and 4).

## Results

**Fig. 2:**
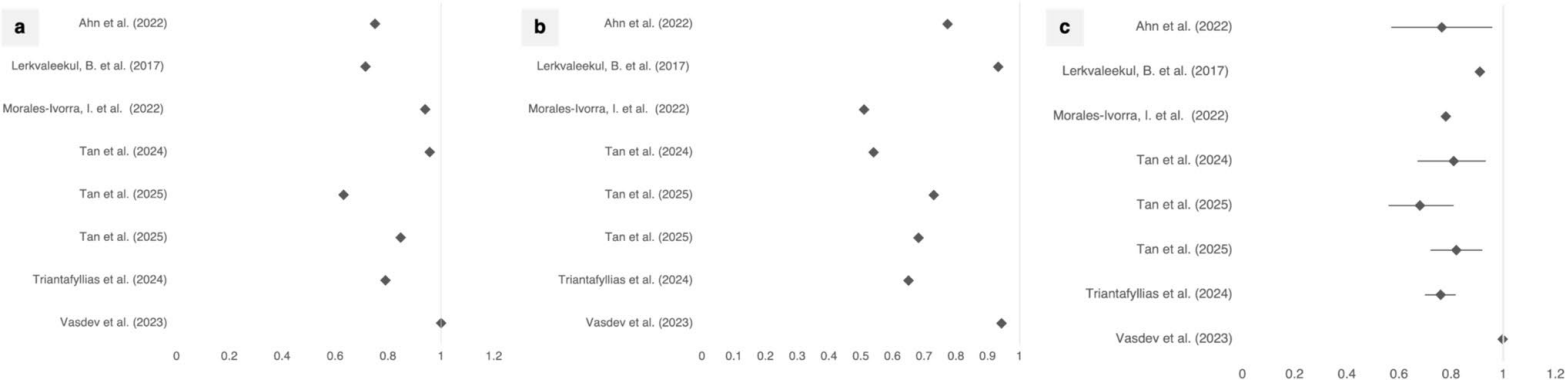
Graphical representation of **a** sensitivity, **b** specificity, and **c** area under the curve (AUC) derived from receiver operating characteristic (ROC) analyses. Studies reporting sensitivity and specificity values were included. Values are shown exemplarily for Tmax. Several studies did not use a simple Tmax parameter but instead applied alternative metrics, which are also presented here: Morales-Ivorra et al. used the ThermoJIS, Tan et al. (2024, 2025) applied a composite score, Triantafyllias et al. used HRR, and Vasdev et al. reported the knee–thigh differential. No 95% confidence intervals were reported for sensitivity and specificity in the mentioned studies. For the area under the curve (AUC), 95% confidence intervals were reported in the majority of studies; however, two studies provided only a single AUC value, for which no 95% confidence intervals are shown

From a total of 945 records identified in the databases over the past 25 years, 19 studies met the inclusion criteria and were analysed (Fig. 1). The results regarding the relationship between IRT and joint US are presented in Table 3 and are further explained in the text below. Eight of the 19 included studies reported sensitivity, specificity, and the area under the curve (AUC) derived from receiver operating characteristic (ROC) analyses. These findings are summarized in Fig. 2.

The diseases investigated include RA, PsA, Juvenile Idiopathic arthritis (JIA), SpA, gout, osteoarthritis (OA), see Table 4 for comparison. The majority of the studies focused on patients with RA (*n* = 13) or mixed disease cohorts (*n* = 4). Two studies included patients with other inflammatory arthritis associated diseases (juvenile idiopathic arthritis (JIA), *n* = 1 and PsA, *n* = 1). Only one study investigated patients under 18 years of age, while all others focused on adults (further information to study populations in Table 4). The sample sizes of the studies ranged from small pilot cohorts to larger datasets.

In all included studies, joint US served as the reference standard (see Table 5 for comparison of methodology). No studies comparing IRT with MRI and bone scintigraphy were identified in the systematic search. While methods were used in these studies varied (see Table 5), most of them employed standardized room conditions, acclimatization phases, and manual region-of-interest (ROI) selection, whereas some applied algorithm-based segmentation. Subtle but potentially significant differences exist in the processing of temperature parameters, contributing to heterogeneity in the literature. For example, Tan et al. used composite scores to assess inflammatory activity across multiple joints [89, 96]; Triantafyllias et al. developed a new thermographic parameter during hotspot identification [79]. Morales-Ivorra [78] and Umapathy et al. [97] process raw data using machine learning and advanced algorithms to evaluate inflammatory activity (further information to study populations in Table 5).

The risk of bias was assessed using the QUADAS-2 assessment tool as recommended by the PRISMA guidelines [80, 81], see Table 6. This revealed potential sources of bias, for example, due to small cohort sizes [83, 84] or the exclusion of significant comorbidities (e.g., osteoarthritis [79]). None of the identified studies used a predefined cut-off, which carries the risk of overestimating test performance.

## Overview of the included studies

### Studies focusing on knee joints

The following section summarizes the results of studies evaluating IRT and joint US in the assessment of knee arthritis, with particular emphasis on their diagnostic potential and accuracy.

Ahn et al. studied 30 patients with swollen knees who were scheduled for arthrocentesis at a tertiary hospital [82]. All patients had knee effusion and a variety of diagnoses, including RA, SpA, OA, and other conditions. The patients underwent IRT, joint US, and arthrocentesis.

Power Doppler (PD)-positive joints, which are indicative of hypervascularization and synovitis, exhibited significantly higher values across all temperature parameters (all; *p* < 0.05), e.g. Tavg 32.1 °C vs 29.1 °C. White blood cell counts correlated with PD positivity (*p* = 0.010)), though no association was observed with thermographic parameters. ROC curve analysis indicated demonstrated good diagnostic performance of Tmax, Tmin, and Tavg for identifying PD positivity (AUC 0.764–0.790). For instance, Tavg demonstrated a sensitivity of 87.5% and a specificity of 68.5% at a cut-off of 30.1 °C (AUC = 0.770, 95% CI 0.581–0.959) [82].

In a subsequent study focusing on knee joints in arthritis, Vasdev et al. compared 50 patients with RA to 50 age- and sex-matched healthy controls recruited from hospital staff [98]. In contrast to the previous protocol, IRT images were obtained from the lateral aspect of the knee with the joint positioned at 90° flexion. To establish a control, a circle of equal radius was positioned on the lateral mid-thigh. Tavg values were measured for each ROI, and a knee–thigh differential (Tavg knee – Tavg thigh) was calculated [98]. Tavg was higher in RA patients than in in the control group (32.7  °C (95% CI 32.37–33.0) vs 31.5  °C (95% CI 31.24–31.74), *p* < 0.05). A significant difference in knee–thigh differential was observed (1.08  °C (95% CI 0.91–1.99) vs − 0.52  °C (95% CI − 0.66 to − 0.37), *p* < 0.05), but no significant association with PD grade was found [98]. ROC analysis showed an AUC of 0.998 (*p* = 0.001), with sensitivity 100% (95% CI 92.5–100%) and specificity 94.4% (95% CI 84.3–98.8%). Notably, 86% of patients were classified as PD grade II, indicating relatively high disease activity [98].

IRT and US of the knee joints were also evaluated in the work of Umapathy et al., including 30 RA patients and 30 healthy controls [97]. IRT data were automatically segmented using the RIBS (Regional Isotherm-Based Segmentation) algorithm to detect hotspots within the ROI on grey-scale images. Temperature parameters and statistical features (mean, variance, skewness, kurtosis) were extracted from the segmented images and compared between RA patients and controls [97]. IRT revealed significant differences between RA patients and controls in extracted parameters and skin temperature (all *p*-values < 0.01). Temperature parameters demonstrated a strong correlation between biochemical markers (ESR: erythrocyte sedimentation rate, CRP: C-reactive protein) and clinical scores (DAS28: disease activity score 28, HAQ: health assessment questionnaire). The correlation was significant at the *p* < 0.01 level. The presence of synovitis, as indicated by GS- (effusion) and PD-positivity (perfusion, color fraction), also demonstrated a correlation with CRP, ESR, DAS28, and HAQ. A direct comparison between IRT and joint US was not performed [97].

Tan et al. investigated 190 knees from 95 patients with rheumatoid arthritis to assess the correlation between thermographic parameters (Tmax, Tmin, and Tavg) and ultrasound findings of knee arthritis [95]. Higher Tmax, Tmin, and Tavg values were significantly associated with joint inflammation detected by grayscale (GS) and power Doppler (PD) ultrasound (GS: correlation coefficients ranged from 0.27 to 0.49, *p* < 0.01; PD: correlation coefficients ranged from 0.21 to 0.43, *p* < 0.05). In addition, receiver operating characteristic (ROC) analyses were performed. The area under the ROC curve (AUC) for Tmax, Tmin, and Tavg in identifying a PD score > 0 ranged from 0.63 to 0.82 [95].

In summary, findings for the knee joint indicate that PD-positive joints were associated with elevated temperature parameters, even though grading of inflammation was not feasible [82, 95]. Moreover, the use of thermal imaging has proven effective in distinguishing RA-affected knees from those of healthy individuals [98]. Automatic segmentation of IRT further offers a practical approach, producing results comparable to joint US [97].

### Wrist and finger joints

Lerkvaleekul et al. compared IRT and joint US findings of the wrist in 46 patients with JIA and 15 healthy controls [86]. JIA patients were categorized as having inactive or active arthritis. The latter category was further subdivided into mild and moderate-to-severe disease based on physical examination. Both Tmax and Tavg in the ROI were found to be significantly higher in the active arthritis group compared to the inactive patients and controls (for example Tavg: Healthy controls 30.27 °C ± SD 0.67; Arthritis 31.37 ± SD 1.49, *p* < 0.05). In subgroup analyses, Tavg and Tmax were highest in the moderate-to-severe group, differing significantly from all other groups, including mild arthritis (*p* < 0.05) [86]. No significant differences were found between mild arthritis and inactive disease.

ROC curve analysis was performed to define a cut-off between healthy controls and the moderate-to-severe arthritis group. For instance, the AUC was 0.93 Tavg > 31 °C (sensitivity 85.7%, specificity 80.0%). For the joint US, the AUC was 0.87, with a sensitivity of 83.3% and specificity of 81.3%. Due to the limited sample size, correlation analysis between thermal imaging and joint US could not be performed; yet the results seemed comparable [86].

Subsequently, Tan et al. studied 70 wrist joints in RA patients to investigate how IRT and US findings relate to clinical signs of arthritis [90]. Wrists were categorized into four clinical groups: (1) swollen and tender (S1T1), (2) swollen and non-tender (S1T0), (3) non-swollen and tender (S0T1), and (4) non-swollen and non-tender (S0T0). Both swollen-joint groups demonstrated higher thermographic parameters compared to the non-swollen/non-tender group (all *p* < 0.05). There were no observed differences between the non-swollen/tender and non-swollen/non-tender groups. Similar results were observed for joint US (*p* < 0.05). The authors highlighted the high comparability between the two diagnostic techniques; however, explicit correlations between IRT and joint US were not analyzed, though the overall findings were considered similar [90].

The same group also focused on the examination of finger joints. In one study, they evaluated 37 RA patients with at least one swollen or tender joint, documented in a binary manner (tenderness/swelling present = 1, absent = 0) [88]. ROIs were manually placed over the MCPs, PIPs, and wrists. Thermographic parameters (Tmax, Tmin, Tavg, Tmax − min) were significantly higher in PD-positive joints (PD-score > 0) than in PD-negative joints (PD-score = 0) (all *p* < 0.001). Similar significant differences were observed for Grey-scale findings (all *p* < 0.001). No significant associations were found between thermographic parameters and clinical features such as tenderness or swelling. The study did not report sensitivity and specificity [88].

In a separate study, Tan et al. investigated subclinical inflammation in the MCP joints of 42 asymptomatic RA patients, comparing joint US and IRT [92]. Joints with active subclinical synovitis (PD-score > 1 or GS-score > 2) showed significantly higher thermographic parameters (Tmin, Tavg, Tmax; *p* = 0.002–0.003; Pearson’s *r* = 0.447–0.465) [92].

Furthermore, the research group expanded its studies to include the detection of subclinical disease activity. They examined the right wrists of 87 RA patients, categorizing them into asymptomatic and symptomatic groups (tender and/or swollen) [91]. Significant differences were found for all thermographic parameters between symptomatic and asymptomatic groups, for example, Tavg 32.0 ± 1.6 vs. 31.0 ± 1.5 (*p* = 0.006). GS and PD scores were higher in symptomatic wrists compared to asymptomatic wrists. Among asymptomatic wrists, those with abnormalities in joint US showed elevated thermographic parameters compared to those without abnormalities, e.g., Tavg 31.5 ± 1.2 vs. 30.3 ± 1.7 (*p* = 0.007) [91]. These results further support the utility of IRT in detecting subclinical synovitis.

In another study, Tan et al. investigated whether summed thermographic parameters (Tmax, Tmin, Tavg) could be used to predict sonographic abnormalities of the bilateral metacarpophalangeal (MCP) joints [96]. To this end, all five MCP joints were examined bilaterally in 81 patients with rheumatoid arthritis, and thermographic parameters were assessed for each joint and additionally summed across joints. They found that higher summed Tmax, Tmin, and Tavg values were significantly correlated with joint inflammation detected by GS and PD US (GS: correlation coefficients ranged from 0.27 to 0.49, *p* < 0.05; PD: correlation coefficients ranged from 0.45 to 0.52, *p* < 0.05). Furthermore, ROC-curve analysis of summed thermographic temperature values for the prediction of a summed PD score ≥ 1 across bilateral MCP joints yielded areas under the curve (AUCs) ranging from 0.80 to 0.82[96].

Tan et al. also developed a composite Total Temperature Score to detect subclinical synovitis. They assessed subclinical disease activity in 80 RA patients using IRT and joint US [93]. Patients were stratified by DAS28 into remission/low disease activity (< 3.2) and moderate-to-high disease activity (> 3.2). US examinations included the MCP, PIP, wrist, elbow, and knee joints. The joints most frequently showing PD positivity were the wrists (62.7%), followed by the second MCPs (37.0%), third MCPs (33.8%), fourth MCPs (24.8%), and fifth MCPs (20.9%) (all *p* < 0.05). In these joints, higher composite Total temperature scores were associated with PD-positivity scores (all; *p* < 0.05). ROC curve analysis established cut-off values for total IRT parameters, with Total Tmax achieving a sensitivity of 95.7% and a specificity of 54.5% for identifying PD-positive joints [93].

A different strategy for detecting synovitis was employed by Morales et al. They developed a machine learning–based Thermographic Joint Inflammation Score (ThermoJIS) to detect arthritis [78]. The study included a large development cohort of 449 participants (development set), comprising patients with RA, PsA, undifferentiated arthritis, OA, and healthy controls. For all patients with rheumatologic diseases, joint US of both hands (wrists, MCPs, and PIPs) was used as the reference standard, with GS and PD abnormalities combined into sum scores. Thermographic images were acquired under non-standardized conditions, intentionally reflecting real-world data. ROIs were automatically defined by an algorithm detecting major intensity variations in the images, and their thermal features were linked to the PD sum score to evaluate associations with synovitis. Data from this development set were used for training and tuning of the machine learning model. Using these data, ThermoJIS was generated [78].

In a validation cohort of 146 RA patients, ThermoJIS was tested for detecting active synovitis, with joint US again serving as the gold standard. ThermoJIS showed moderate correlations with grey-scale synovial hypertrophy (*r* = 0.49, *p* < 0.001) and Power Doppler (*r* = 0.51, *p* < 0.001). Sensitivity was 91% and specificity was 51%. Importantly, ThermoJIS also detected subclinical activity in RA patients in remission (DAS28-CRP < 2.6).

To enhance specificity, the authors proposed classifying a range of scores as “indeterminate,” which adjusted sensitivity and specificity to 87% and 82%, respectively. However, 43% of the validation cohort fell into this indeterminate range, which the authors noted could limit the practical utility of the method [78].

Interestingly, in a subsequent study on the same validation cohort, the authors combined ThermoJIS with the patient’s global assessment (PGA), creating ThermoDAI, or with PGA and CRP, creating ThermoDAI-CRP [87]. Both indices demonstrated stronger correlations with active synovitis detected by joint ultrasound than ThermoJIS alone (e.g., Spearman’s *r*: ThermoDAI-CRP vs. PD sum score = 0.61; ThermoJIS vs. PD sum score = 0.51) [87]. ThermoDAI and ThermoDAI-CRP also correlated strongly with established disease activity scores (CDAI, SDAI, DAS28-CRP) and showed comparable sensitivity and specificity for detecting active synovitis [87].

Tan et al. also investigated the relationship between disease activity (operationalized using DAS28) in 37 RA patients with composite IRT and US scores [89]. For the IRT, they determined the lowest measured temperature of a patient’s joint and then calculated the difference between Tmax, Tmin, and Tavg for each joint examined. The differences for each joint were then added together to form the composite scores MAX, MIN, and AVG. They also formed composite scores for the US (total PD and total GS). In addition, combined scores were also formed from the results of the IRT and US (MAX (PD), MIN (PD), and AVG (PD)). The statistical correlation with DAS28 was then examined [89].

This showed that both the combined scores MAX (PD) and AVG (PD) correlated significantly with the DAS28 (for example Pearson correlation coefficient MAX (PD) (95% CI), 0.393 (0.079, 0.636), *P* = 0.016). IRT and US alone did not achieve a significant correlation. This indicates the value of using both diagnostic methods in parallel [89].

In previous studies, cutaneous surface temperature was typically measured once and compared with joint US. Capo et al. (2015) employed a different approach, comparing 10 patients with psoriatic arthritis (PsA) and 11 healthy controls [83]. ROIs were manually defined on the dominant hand (MCP, PIP, DIP), and a thermographic baseline was established. Standardized isometric contractions of the dominant hand muscles were then performed using a dynamometer, with various temperature parameters recorded over 5 min. PsA patients exhibited significantly higher temperature values (*p* < 0.0025) and faster temperature fluctuations compared to controls [83]. Some temperature parameters correlated with PD-defined disease activity. However, the small sample size (6 with active and 4 with inactive disease) limited the subgroup analysis, allowing only qualitative conclusions [83].

In a subsequent methodological study, Capo et al. (2018) compared 10 RA patients with 11 healthy controls [84]. In contrast to the findings of the 2015 PsA study, RA patients demonstrated lower dynamic temperature values compared to the control group. A joint US was also performed on RA patients to facilitate a comparison with the findings from IRT. Due to the limited sample size, only qualitative conclusions could be drawn, and no further details were provided [84].

Overall, when comparing the ability of IRT and US to detect arthritis in wrist joints, both modalities demonstrated similar performance in head-to-head ROC analyses [86]. IRT successfully identified both symptomatic and asymptomatic inflamed wrist joints, with inflammation defined by ultrasound criteria [90, 91]. Similar findings were reported for asymptomatic MCP joints [92]. Moreover, the application of automated image analysis techniques has shown promising results, as demonstrated by Morales-Ivorra et al. [78, 87], while Capo et al. highlighted the potential of dynamic thermal measurements as a valuable complementary approach [83, 84]. The combination of IRT and US could measure disease activity even more accurately than either method alone [89].

## Other ioints (e.g., elbow, foot) and mixed disease spectra

Studies assessing the diagnostic performance of thermography for the other joints, such as the elbow or the foot joints, and mixed disease spectra are limited.

Tan et al. evaluated elbow IRT in 30 RA patients, performing bilateral examinations [94]. ROIs were manually selected from all four sides of the elbow joints, and Tmax, Tmin, and Tavg were measured. The composite scores were generated by summing Tmax values from all four aspects to create a total Tmax score (MAX). The same procedure was applied for Tmin and Tavg (MIN and AVG). GS- and PD- subscores were also summed. A notable correlation was identified between the composite thermographic scores and PD scores in both elbows. For instance, the correlation coefficient (95% CI) for MAX was 0.47 (0.12–0.70). Thermographic scores and grey-scale findings have been found to correlate only with the right elbow (*p* < 0.05) [94].

Moreover, Gizinska et al. examined the dorsal feet of 81 RA patients with pain and stiffness, compared to 39 healthy controls [85]. RA patients exhibited significantly higher mean ROI temperatures than controls (*p* < 0.05). However, ultrasound-detected inflammation generally did not correspond with elevated temperature parameters, except in the right MTP1 (metatarsophalangeal joint), left MTP2, and two ROIs (right ROI 1, left ROI 2), where *p* < 0.05 [85].

Triantafyllias et al. assessed multiple joints in 75 patients with mixed disease spectra, including RA, PsA, SpA, and gout, as well as 70 healthy controls [79]. A novel parameter, the hotspot/ROI ratio (HRR), was defined using a k-means clustering algorithm and compared with PD grades I–III. Fingers, wrists, elbows, knees, and ankles were examined, with analyses conducted both for the overall patient group and for disease-specific subgroups. Tmin/max/avg were significantly higher in PD-positive joints compared to controls. HRR showed the same pattern (*p* < 0.0001) and, together with Tavg, distinguished PD-positive from PD-negative joints (HRR: 0.313 ± 0.031 vs 0.103 ± 0.027, *p* < 0.0001; Tavg: 31.43 ± 0.32 vs 30.88 ± 0.31, *p* = 0.026). HRR correlated moreover with semi-quantitative PD scores, except between PDUS grade I and II (*p* = 0.417). ROC analysis of HRR yielded an AUC of 0.76 (95% CI 0.70–0.82) with 79% sensitivity and 65% specificity. The best performance was seen at the wrist (AUC 0.91, 95% CI 0.84–0.98; sensitivity 83%, specificity 88%) and large joints (AUC 0.87, 95% CI 0.80–0.94; sensitivity 82%, specificity 79%) [79].

The elbow and foot regions have been investigated to a limited extent. While studies on the elbow emphasize the potential diagnostic value of IRT [94], investigations of the feet using the applied methodology have shown little clinical benefit [85]. Further research, potentially with modified study designs, appears necessary. Notably, Triantafyllias et al. demonstrated the strong diagnostic performance of IRT [79]; using HRR-based processing of temperature parameters, they were able to identify associations with ultrasound-derived inflammation grades [79]. Nevertheless, studies involving larger cohorts and more diverse disease populations remain scarce [78, 79].

## Discussion

This systematic review summarizes the evidence on medical IRT in rheumatology, focusing on studies that used joint US as an established reference [100, 101, 111]or evaluated both modalities concurrently. To our knowledge, it is the first systematic review to directly compare IRT with an established diagnostic modality and among the few works assessing the diagnostic value of IRT in rheumatology practice in general [41, 71].

The reported studies suggest that IRT can distinguish healthy joints from arthritic joints, with results generally consistent with US [79, 86, 98]. Additionally, like joint US, IRT appears capable of detecting both active and inactive disease [79, 93].

The current literature on IRT and its relationship to joint US is highly heterogeneous regarding preparation of the patient and data processing following data acquisition (see Table 5). While most studies applied standardized preparation protocols, still differences in factors such as room temperature, acclimatization time, camera placement, and especially ROI selection limit the comparability of results [112]. The use of international guidelines, such as those from the European Association of Thermology [102, 103], and standardized approaches like the Glamorgan Protocol for ROI selection [104], may contribute to enhancing consistency across studies. In two studies, these guidelines had already been implemented [79, 85].

As described before, the methods used to evaluate thermographic images vary significantly across studies. Some studies employed relatively straightforward metrics, such as the average temperature of an ROI [82] or its ratio to the average temperature of an independent skin area [98]. Another approach was the use of composite score of multiple joints [89]. Others applied even more sophisticated approaches, including automatic ROI selection with machine learning [78] or the hotspot-to-ROI ratio (HRR), which identifies ‘hotspots’ in the ROI via clustering and correlates strongly with joint US results [79].

Several studies had small sample sizes (20–27 subjects) [83, 84], limiting the strength of their conclusions. The majority of the research focused on RA (13 of 19 studies), with only four studies including mixed patient groups. In some cases, important differential diagnoses such as osteoarthritis were excluded [79], despite its high prevalence, impact on disability, and association with increased joint temperature [113, 114].

The aforementioned limitations and methodological differences may contribute to the wide reported range of sensitivity and specificity (sensitivity for detecting arthritis ranged from 79 to 100%, specificity from 51–94%, as depicted in Fig. 2). This broad range underscores the need for further standardization [41, 71]. Findings of good specificity such as those by Triantafyllias et al. demonstrate the potential utility in routine clinical practice [79].

On the other hand, the methodological heterogeneity in IRT has also led to the development of multiple approaches that could enhance its diagnostic application. Some methods, such as machine learning–based approaches [78] or cumulative temperature scores [93], show high sensitivity for detecting disease activity. Others, such as the HRR parameter demonstrated high specificity [79]. Combining these approaches in a stepwise diagnostic framework could be valuable in different clinical scenarios [89]. Additionally, dynamic test procedures, as used by Capo et al. [83, 84], may complement static assessments in cases with inconclusive findings in the future.

Integrating IRT with joint US has the potential to enhance diagnostic accuracy and efficiency. Given the limitations of joint US, including high examiner dependency and personnel intensity [50], thermography offers a valuable complement, by enabling rapid, examiner-independent assessment of multiple joints, potentially even by non-medical personnel.

Overall, thermography appears to be a promising adjunct for diagnosing and monitoring arthritis, providing high sensitivity and specificity depending on the method used. Its performance seems to be particularly strong in larger joints, such as the knee and wrist [79, 98], while results in smaller joints, like the MCPs, are also encouraging [92, 96]. Other small joints, such as those of the feet, showed disappointing results; however, this was observed in only a single study. Further research in this area is needed [85].

Several of the included studies were conducted by the same author group (Tan et al., see Table 3), reflecting the current structure of the research field. While studies were identified through a predefined, comprehensive search strategy independent of authorship, this concentration may limit the independence and generalizability of the evidence and should be considered when interpreting the findings.

To minimize risk of bias in future studies, it also appears important to test predefined cut-offs in as realistic and diverse study populations as possible, encompassing a variety of disease conditions, in order to better estimate performance in routine clinical practice.

In the future research is needed to assess the applicability of thermography in outpatient settings for both diagnosis and follow-up, and to evaluate its reliability in supporting treatment decisions compared with joint US. Looking forward, this approach could complement telemedicine-based assessments [72] and may provide a valuable tool to support AI-assisted patient care within automated diagnostic or therapeutic workflows, potentially enhancing both efficiency and the accuracy of clinical decision-making [74, 75].

Moreover, its utility in conditions other than RA and in paediatric populations warrants further investigation.

## Abbreviations

ARD: Autoimmune rheumatic diseases
AUC: Area under the curve
GS: Grey-scale joint ultrasound
IRT: Infrared thermography
JIA: Juvenile idiopathic arthritis
MCP: Metacarpophalangeal joint
MTP: Metatarsophalangeal joint
OA: Osteoarthritis
PD: Power doppler ultrasound
PIP: Proximal interphalangeal joint
PsA: Psoriatic arthritis
RA: Rheumatoid arthritis
ROC: Receiver operating characteristics
ROI: Region of interest
SpA: Spondylarthritis
Tavg: Average temperature
ThermoJIS: Thermographic joint inflammation score
Tmax: Maximum temperature
Tmin: Minimum temperature
US: Ultrasound
